# Antioxidant and Hepatoprotective Effect of Aqueous Extract of Germinated and Fermented Mung Bean on Ethanol-Mediated Liver Damage

**DOI:** 10.1155/2013/693613

**Published:** 2012-12-24

**Authors:** Norlaily Mohd Ali, Hamidah Mohd Yusof, Kamariah Long, Swee Keong Yeap, Wan Yong Ho, Boon Kee Beh, Soo Peng Koh, Mohd Puad Abdullah, Noorjahan Banu Alitheen

**Affiliations:** ^1^Department of Cell and Molecular Biology, Faculty of Biotechnology and Biomolecular Sciences, University Putra Malaysia, 43400 Serdang, Selangor, Malaysia; ^2^Department of Bioprocess Biotechnology, Malaysian Agriculture Research Development Institute, 43400 Serdang, Selangor, Malaysia; ^3^Institute of Bioscience, University Putra Malaysia, 43400 Serdang, Selangor, Malaysia; ^4^Department of Bioprocess Technology, Faculty of Biotechnology and Biomolecular Sciences, University Putra Malaysia, 43400 Serdang, Selangor, Malaysia

## Abstract

Mung bean is a hepatoprotective agent in dietary supplements. Fermentation and germination processes are well recognized to enhance the nutritional values especially the concentration of active compounds such as amino acids and GABA of various foods. In this study, antioxidant and hepatoprotective effects of freeze-dried mung bean and amino-acid- and GABA-enriched germinated and fermented mung bean aqueous extracts were compared. Liver superoxide dismutase (SOD), malondialdehyde (MDA), ferric reducing antioxidant power (FRAP), nitric oxide (NO) levels, and serum biochemical profile such as aspartate transaminase (AST), alanine transaminase (ALT), triglycerides (TG), and cholesterol and histopathological changes were examined for the antioxidant and hepatoprotective effects of these treatments. Germinated and fermented mung bean have recorded an increase of 27.9 and 7.3 times of GABA and 8.7 and 13.2 times of amino acid improvement, respectively, as compared to normal mung bean. Besides, improvement of antioxidant levels, serum markers, and NO level associated with better histopathological evaluation indicated that these extracts could promote effective recovery from hepatocyte damage. These results suggested that freeze-dried, germinated, and fermented mung bean aqueous extracts enriched with amino acids and GABA possessed better hepatoprotective effect as compared to normal mung bean.

## 1. Introduction

Liver is a pivotal inflammatory organ that, involved in metabolism, storage, and excretion of metabolites. There are considerable numbers of hepatotoxins that have been reported to cause a liver damage such as ethanol, paracetamol, and carbon tetrachloride [1–5]. The mice model of liver injuries induced by various hepatotoxins showed similar trend but with slight variations such as increased membrane permeability, lipid peroxidation, and cell death which was comparable to development of chronic hepatic disease in humans. Upon stimulation from various hepatotoxins, Kupffer cells which release proinflammatory mediators such as NO and Interferon-gamma (IFN-*γ*) will eventually result in accumulation of reactive nitrogen species (ROS). ROS has been shown to cause lipid peroxidation and membrane degradation which will generate liver damage and inflammation [1, 3, 5, 6]. Natural sources of antioxidantssuch as green tea has been reported to increase the level of SOD and FRAP in cytoplasm of rat's liver which reverted the injury effect close to normal [7]. Polyphenols, flavonoids, and anthocyanins have been suggested to exert strong antioxidant activity, which contribute to the protective effect of against liver injury in rats [4, 7].

Mung bean (*Vignaradiata*), which is mainly cultivated in East Asia and South Asia regions contains rich source of protein, essential amino acids, minerals, vitamins, and fibers. It has been renowned of having multinutritional values as well as medicinal properties. Earlier studies have demonstrated that mung bean can act as an antioxidant [2, 8], liver protection [9] and antidiabetic agent due to its low glycemic index [10, 11]. Recently, it was reported that the ethanolic extract of mung bean exhibits antiinflammatory response by decreasing the proinflammatory cytokines in mouse macrophages [5, 12]. Germination and fermentation have been well associated with the elevated amount of antioxidants and GABA content [13–16]. GABA (*γ*-amino butyric acid) is a nonprotein amino acids that acts as a neuron inhibitors in mammals, which can be extracted from plant. Numerous studies have proclaimed that the roles of GABA as an antihypertension, anticancer, and antiinflammatory agents and its other healthful benefits [17–19]. These aspects have stimulated the interest to generate GABA-enriched natural products. Fermented soybean product, GABA-Tempeh, is a traditional food that contains abundance of oligopeptides and free amino acids (mainly GABA) which contribute to lower level of cholesterol in plasma [18]. This effect can be correlated with liver function as lipid metabolism in body. Therefore, by undergoing germination and fermentation processes, freeze-dried mung bean aqueous extracts could contribute to liver protective effect and other healthful benefits. Wu et al. [9] were the first to report the hepatoprotective effects of mung bean. Their study compared the histological and biochemistry changes of acetaminophen-induced liver injury and the ameliorate properties of different Taiwan's legumes such as adzuki bean, black bean, rice bean, and mung bean. Mung bean aqueous extract was identified to exhibit the best hepatoprotective effects among the legumes against liver injury agent, acetaminophen. 

To date, no *in vivo* test has been conducted to assess the effect of freeze-driedgerminated and fermented mung bean aqueous extracts on animal model. The purposes of this study wereto compare the *in vivo* antioxidant enzymes content and hepatoprotective effects of freeze-dried normal mung bean, nutrient-enriched germinated, and fermented mung bean aqueous extracts on ethanol-induced liver damage mice model. This study also aimed to establish the correlation between the effects of fermentation and germination on amino acids and GABA level of mung bean and the hepatoprotective properties of the extracts.

## 2. Materials and Methods

### 2.1. Materials

Hypoxanthine, xanthine oxidase, superoxide dismutase, Folin-Ciocalteu reagent, aluminium chloride, sodium nitrate, ascorbic acid, and gallic acid were purchased from Sigma-Aldrich (USA). All solvents used were either of analytical reagent or HPLC grade. Griess reagent was from Invitrogen (USA). The *Rhizopus* sp. strain of 5351 inoculums was obtained from MARDI's (Malaysian Agricultural Research and Development Institute) culture collection center. Milk thistle extracts containing 80% of silybin was obtained from Lipa Pharmaceutical Pty. Ltd. (Australia).

### 2.2. Animals

Male Balb/c mice of 8–10 weeks old weighing 20–25 g were maintained under standard condition of temperature (22 ± 5°C) and humidity in animal house with 12 h of light/dark cycle. Animals were provided with food and water ad libitum. Experiments were strictly conducted and approved by Animal Care and Use Committee, Universiti Putra Malaysia, (Ref: UPM/FPV/PS/3.2.1.551/AUP-R2).

### 2.3. Plant Material

Mung bean (*Vignaradiata*) seeds were purchased from the local store in Selangor. The mung bean seeds were allowed to undergo solid-state fermentation base on our previous method [20] and germination process prior to extraction. For fermented mung bean [20], about 1000 g of dehulled mung bean seeds were soaked in cold water at room temperature for 18 h. Soaked mung beans were washed thoroughly and steamed for 40 minutes. After that, steamed seeds were cooled to room temperature and subsequently mixed with *Rhizopus* sp. strain of 5351 inoculums. From our previous preliminary studies (data not shown), mung bean seeds were screened with different *Rhizopus* sp. strains (5346, 5347, 5351, 5375, 5376, 5377, 5408, and 5410). The results revealed that *Rhizopus* sp. strain 5351 yielded the highest total amino acids and GABA content in fermented mung bean after 48 h of incubation at 30°C. Following this, the hepatoprotective effects of fermented mung bean inoculated with 5351 strain were evaluated. The inoculated beans were then packed into perforated plastics and incubated for 48 h at 30°C. Finally, all fermented mung bean seeds were dried and ground into powder prior to water extraction. On the other hand, germinated mung bean seeds was prepared by germinating the mung beans seeds inside the container *Anaerocult A *supplied with CO_2_ gas for up to 72 h. Germinated seeds were then allowed to dry until constant moisture content was obtained and ground into powder prior to water extraction. For control, mung bean seeds were directly ground into powder without prior fermentation or germination.

Finely ground powder was then extracted using deionised water (1 : 20 ratio) at 25°C for 30 minutes and placed in an incubator shaker at 300 rpm for 30 minutes under room temperature. Mixture was then centrifuged for 5 minutes at 10,000 rpm andthe supernatant was collected. Supernatants were furthersubjected to freeze-dry at operating temperature of −50°C (yield 25%, w/w). The freeze-dried powder was stored at 4°C. The assays were performed according to [20–22] with slight modifications.

#### 2.3.1. GABA and Amino Acids Determination

The freeze-dried powder was dissolved in distilled water and filtered through 0.2 *μ*m syringe filter prior to UPLC analysis. The derivatization process was done by mixing 70 *μ*L of AccQ-Tag Ultra borate buffer with 10 *μ*L of filtered extracts solution, followed by adding 20 *μ*L of AccQ Fluor reagent in 1.5 mL eppendorf tube. All analyses were performed on a Waters Acquity UPLC system, comprised of a binary solvent manager, a sample manager fitted with 2 *μ*L sample loop and UV-PDA detector set at 260 nm. The data were analyzed using Waters Empower 2 software. Acquity UPLC AccQ-Tag Ultra Column (2.1 mm i.d. × 100 mm × 1.7 *μ*m particle size) was used for the determination of GABA and amino acids profile. The mobile phase used was AccQ-Tag Ultra Eluent A for mobile phase A and AccQ-Tag Ultra Eluent B for mobile phase B. The gradient condition was: 0–0.54 minutes, 0–0.1% B; 0.54–5.74 minutes, 0.1–9.1% B; 5.74–7.74 minutes, 9.1–21.2% B; 7.74–8.8 minutes, 21.2–59.6% B; 8.8–11 minutes, 59.6–0.1% B, and finally, reconditioning the column with 0.1% B with isocratic flow for 2.1 minutes after washing column with 59.6% B for 0.30 minutes. The flow rate was set at 0.7 mL/minutes and the injection volumes for all samples and standards were 1.0 *μ*L. The column temperature was set at 55°C according to [20, 23].

### 2.4. *In Vivo* Hepatoprotective Effect-Ethanol Induced Hepatotoxicity in Mice

Total of 72 Balb/c mice were randomly distributed into eight groups (*n* = 8). Hepatoprotective effects of freeze-dried mung bean and fermented and germinated mung bean aqueous extracts were assessed in ethanol-induced liver damage animal model. Mice were pretreated orally with ethanol and plant aqueous extracts individually for up to 21 days. The experiment was designed as follows.

*Group  1*. Normal group, mice (p.o.) with 100 *μ*L of normal saline for 14 days.

*Group  2*. Ethanol untreated group, mice (p.o.) with 100 *μ*L of 50% (v/v) of ethanol for 7 days followed by 14 days of 100 *μ*L of 1 X PBS.

*Group  3*. Positive control group, mice (p.o.) with 100 *μ*L of 50% (v/v) of ethanol for 7 days followed by 14 days of 100 *μ*L of silybin (50 mg/kg).

*Group  4*. Low dose treated group, mice (p.o.) with 100 *μ*L of 50% (v/v) of ethanol for 7 days followed by 14 days of 100 *μ*L of mung bean extract (200 mg/kg).

*Group  5*. High dose treated group, mice (p.o.) with 100 *μ*L of 50% (v/v) of ethanol for 7 days followed by 14 days of 100 *μ*L of mung bean extract (1000 mg/kg).

*Group  6*. Low dose treated group, mice (p.o.) with 100 *μ*L of 50% (v/v) of ethanol for 7 days followed by 14 days of 100 *μ*L of germinated mung bean extract (200 mg/kg).

*Group  7*. High dose treated group, mice (p.o.) with 100 *μ*L of 50% (v/v) of ethanol for 7 days followed by 14 days of 100 *μ*L of germinated mung bean extract (1000 mg/kg).

*Group  8*. Low dose treated group, mice (p.o.) with 100 *μ*L of 50% (v/v) of ethanol for 7 days followed by 14 days of 100 *μ*L of fermented mung bean extract (200 mg/kg).

*Group  9*. High dose treated group, mice (p.o.) with 100 *μ*L of 50% (v/v) of ethanol for 7 days followed by 14 days of 100 *μ*L of fermented mung bean extract (1000 mg/kg).

At the end of the experimental period, mice were sacrificed by cervical dislocation. Blood serum was obtained via cardiac puncture and subjected to serum biochemistry analysis and liver was immediately collected. Weight of liver was recorded and expressed as a relative organ weight [24].

### 2.5. Serum Biochemistry

Activities of blood serum marker enzyme including alanine transaminase (ALT), aspartate aminotransferase (AST), triglyceride (TG), and total cholesterol were measured using biochemical analyzer (Hitachi 902 Automatic Analyzer) and adapted reagents from Roche (Germany).

### 2.6. Liver Histopathological Evaluation

Liver was removed, fixed in 10% formalin solution, embedded in paraffin, sectioned into 4 microns thickness, and stained with haematoxylin and eosin (H&E) for assessment of histopathological alterations. Histopathological changes of stained livers were observed under bright-field microscope. Assessment of liver was graded based on vascular and necrotic changes according to [25]. Vascular changes include vessel congestion, leakage of erythrocytes into surroundings, and hematoma formation. Necrotic changes show the appearance of necrosis, fibrosis, and cell regeneration. No change (no distinguishable change, 0%); mild change (30%); moderate change (31–60%); severe change (61–90%); very severe change (91–100%).

### 2.7. *In Vitro* Antioxidants of Liver Homogenate Evaluation

Mice liver were meshed in ice-cold PBS and homogenized before centrifuged at 2000 rpm for 5 minutes at 4°C. Supernatant was collected and subjected to different assays including superoxide dismutase (SOD) [26], malondialdehyde (MDA) [27], ferric reducing antioxidant power (FRAP) [28] and nitric oxide (NO) assay [29]. 

#### 2.7.1. Determination of Superoxide Dismutase (SOD)

Briefly, SOD was determined following the method of evaluating the inhibition of the reduction of nitro blue tetrazolium (NBT) of liver homogenates. Briefly, sample was added with 0.1 mol/L EDTA, 0.15 mg/mL sodium cyanide, 1.5 mmol/L NBT, 0.12 mmol/L riboflavin, and 0.067 mol/L phosphate buffer to a final volume of 300 *μ*L. The reduction was measured at 560 nm and percentage of SOD inhibition as compared to the blank was determined. One unit of SOD was calculated by the amount of protein needed to achieve the 50% inhibition and hence expressed as unit SOD/mg protein.

#### 2.7.2. Determination of Malondialdehyde (MDA)

Liver peroxidation was detected by measuring thiobarbituric acid-reactive substance (TBARS). In brief, aliquot of 100 *μ*L liver homogenate was diluted with 400 *μ*L of PBS (8.1 g NaCl, 2.302 g Na_2_HPO_4_, and 0.194 g NaH_2_PO_4_/L) and mixed with 12.5 *μ*L butyhydroxytoulene (BHT, 8.8 mg/mL) and 250 *μ*L trichloroacetic acid (TCA, 30%). The mixture was vortexed and kept on ice for 2 h. Next, mixture was centrifuged at 2000 g for 15 min. Supernatant obtained was boiled for 15 min along with 37.5 *μ*L 0.1 M EDTA and 125 *μ*L thiobarbituric acid (TBA, 1%). After mixture has been cooled down to room temperature, the absorbance of pink-colored product was taken at 532 and 600 nm wavelength using ELISA Reader (Bio-tek Instrument, USA). The difference between absorbance was measured and compared to that of the standard malonaldehyde tetramethyl acetal solutions of different concentrations. MDA activity was expressed as nmol MDA/g protein.

#### 2.7.3. Determination of Ferric Reducing Antioxidant Power (FRAP)

The FRAP was determined from reduction of Fe^3+^ to Fe^2+^ according to standard method with some modification. Reagent was prepared by mixing 300 mM acetate buffer (3.1 g C_2_H_3_NaO_2_·3H_2_O and 16 mL C_2_H_4_O_2_), 10 mM TPTZ (2, 4, 6-tripyridyl-*s*-triazine) solution, and 20 mM FeCl_3_·6H_2_O solution in 40 mM HCl. The fresh working solution was prepared by mixing 25 mL acetate buffer, 2.5 mL TPTZ solution, and 2.5 mL FeCl_3_·6H_2_O solution and then warmed at 37°C before using. Aliquot of 150 *μ*L of bioactive extract (5 mg/mL) from mung bean, germinated, and fermented beans was allowed to react with 2850 *μ*L of FRAP solution and shaken vigorously before being incubated in the dark for 30 min. The reading of the colored product (ferrous tripyridyltriazine complex) was taken at 593 nm. The FRAP activity was calculated from the standard FeSO_4_ calibration curve and FRAP value was expressed as *μ*M Fe^2+^/mg protein.

#### 2.7.4. Determination of Nitric Oxide

Briefly, NO production in liver was determined using a calorimetric Griess reaction (Invitrogen, USA). Liver homogenates (100 *μ*L) was loaded onto microtitre plate, followed by 100 *μ*L Griess reagent (1% sulphanilamide and 0.1% N-1-naphthylethylenediamine dihydrochloride in 2.5% polyphosphoric acid). Later, the absorbance was taken at 540 nm wavelength using ELISA Reader (Bio-tek Instrument, USA).

### 2.8. Statistical Analysis

All quantitative measurements were conveyed as mean ± SD Analyses were performed using one-way analysis of variance (ANOVA) and the group means were compared by Duncan test. *P* < 0.05 was considered as statistically significant. 

## 3. Results

### 3.1. GABA and Amino Acids Content

We have previously reported that fermented mung bean contained 7.6 times and 13.2 times higher GABA and amino acids contents as compared to normal dried mung bean powder [20]. Similarly, germinated mung bean also showed an increase in GABA and amino acids concentration by 27.9 times and 8.7 times to 0.502 ± 0.035 g/100 g and 2.092 ± 0.117 g/100 g of dried powder, respectively.

### 3.2. *In Vivo* Hepatoprotective Effect

#### 3.2.1. Effect of Aqueous Extracts on Liver Function Biomarkers

ALT and AST are two biochemical markers normally used for early stage assessment of liver injury.  Table 1 shows that ethanol had significantly raised serum ALT and AST level in mice liver as compared to normal group indicating the incident of liver injury. The serum ALT level was successfully brought down in all posttreatment groups with high doses of mung bean, germinated and fermented mung bean extracts (1000 mg/kg). In contrast, the serum ALT level in all low doses of mung bean extracts (200 mg/kg) treated groupswere continued to rise, indicating that the functions of liver have been compromised. In all extract-treated groups of both concentrations, the serum markers of AST were reduced to lower than the ethanol-attenuated group. Treatment with fermented mung bean at high dose (1000 mg/kg) displayed the highest suppression percentage of serumALT (63.73%) and AST (69.84%) followed by germinated mung bean high dose (1000 mg/kg), 45.25% (ALT) and 47.75% (AST), when compared to ethanol control group.

The above results showed that fermented mung beans at high dose (1000 mg/kg) were able to retain the serum ALT and AST closest to the normal level and has better performance than the standard drug, silybin. 

#### 3.2.2. Effect of Aqueous Extracts on Serum TG and Cholesterol

Another hallmark to confirm the acute alcohol-induced liver injury was indicated by elevated serum TG and cholesterol level. As shown in Table 1, treatment with extracts subsided the boosted level of TG and cholesterol with significant reduction in high dose fermented mung bean (1000 mg/kg) with 38.4% and 23.42%, respectively.

### 3.3. Effect of Aqueous Extracts on the Level of SOD, MDA, FRAP, and NO in Liver Homogenate

The effects of oral administration of mung bean, germinated, and fermented mung bean aqueous extracts on liver antioxidant were shown in Table 2. After being intoxicated with ethanol, a decline in the level of superoxide dismutase (SOD) and ferric reducing antioxidant power (FRAP) was observed in liver injury groups (ethanol-induced) when compared to normal group. Yet, SOD level increased back to normal in all extracts-treated mice with low (200 mg/kg/day) and high doses (1000 mg/kg/day) of mung bean, germinated, and fermented mung beans. On the other hand, MDA and NO levels were markedly increased in ethanol-attenuated liver, hallmarks of lipid peroxidation, and inflammatory response. Significant decrease in MDA and NO production were noticed in all aqueous extract-treated groups. Fermented mung bean was able to reduce MDA level by 3.6 times from 7.17 ± 0.17 to 2.00 ± 0.23 (nmol/g of protein) and NO level by 1.6 times from 14.72 ± 0.75 to 9.03 ± 0.06 (*μ*mol/mg of protein). Meanwhile, it also elevated the SOD enzyme level and FRAP activity by 2.3 and 2.2 times, respectively, which essentially contribute to hepatoprotective effects against free radicals. Thehighest dose of fermented mung bean (1000 mg/kg/day) was found to be the most comparable to normal and standard drug silybingroups.

### 3.4. Histopathological Evaluation

**Figure 1:**
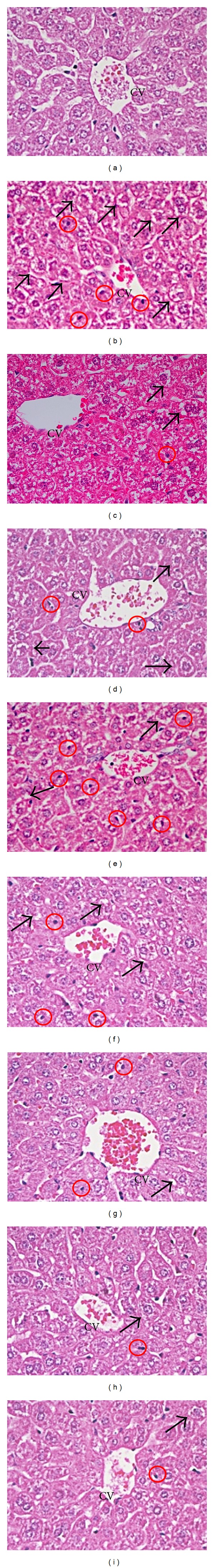
The photomicrographs (40 × 10) of liver section taken from mice. Normal group (a) received saline as a normal control group, shows a normal structure of central vein surrounded by hepatic cells, (b) received saline after being induced with 50% ethanol as a ethanol control group, shows a steatosis and hepatocyte necrosis; (c) received Silybin (50 mg/kg body wt.) after being induced with 50% ethanol; (d) received mung bean (200 mg/kg body wt.) after being induced with 50% ethanol; (e) received mung bean (1000 mg/kg body wt.) after being induced with 50% ethanol; (f) received germinated mung bean (200 mg/kg body wt.) after being induced with 50% ethanol; (g) received germinated mung bean (1000 mg/kg body wt.) after being induced with 50% ethanol; (h) received fermented mung bean (200 mg/kg body wt.) after being induced with 50% ethanol; (i) received fermented mung bean (1000 mg/kg body wt.) after being induced with 50% ethanol. Significant hepatoprotective effects are seen in extracts-treated particularly germinated and fermented mung bean. Arrow indicates a condition of microvesicular steatosis in liver injury, which mainly occurs in ethanol-induced group. Circle indicates hepatocytes necrosis. Centrilobular vein (CV).

Histopathology assessment of liver was performed for all groups.  Figure 1(a) shows that there was no pathological abnormality observed in the liver of normal mice and thus showing the absence of vascular or necrosis changes.  Figure 1(b) shows that ethanol induced severe necrosis changes and substantial changes in liver section such as ballooning, microvesicular steatosis, increase in sinusoidal space (SS) dilation and central vein, and lymphocytes cells infiltration in sinusoids in ethanol-untreated group as compared to normal group. The striking feature observed in ethanol-induced liver was in various stages of cytoplasmic condensation, microvesicular steatosis, and hepatocytes necrosis indicating early phases of liver injury. On the other hand, livers of mice in all aqueous extracts-treated groups showed noticeable recovery from ethanol-induced liver damage when compared to ethanol untreated group with less microvesicular steatosis and hepatocytes necrosis features. Moderate necrosis changes were noticed in all low doses of mung bean extracts-treated groups (Figures 1(d), 1(f), and 1(h)). The high dose of mung bean, germinated, and fermented mung bean aqueous extracts-treated groups illustrated mild necrosis and inflammatory changes, with less severity than changes observed after ethanol administration (Figures 1(e), 1(g), and 1(i)). Reduced degree of sinusoidal and central vein dilations, ballooning, and hepatocytes necrosis were noticed particularly in germinated and fermented mung beanat high dose (1000 mg/kg) (Figure 1(g)).

## 4. Discussion

Ethanol has been reported as an eminent contributor to liver and kidney injury in humans and animals who have been exposed to excess ethanol for a certain period of time [1, 30]. Ethanol metabolism can trigger protein, lipid, and DNA degradation due to free radicalsformation. The result of the present study supports the work of previous published reports using natural extracts to treat ethanol-induced mice, as a model for acute liver disease [31, 32]. Features of ethanol-attenuated hepatocytes include inflammation, apoptosis, and necrosis including cirrhosis. In addition, prolonged exposure to ethanol has been shown to increase the level of TNF-*α*, a proinflammatory cytokines, which in turn can trigger other inflammatory chemokine, explicitly, NO. Decrease in antioxidant defense and elevation of serum markers such as AST, ALT, TG, and cholesterol were also observed [4, 33]. Furthermore, numerous studies have reported the association of antioxidant in the protection against oxidative liver injury [5, 34, 35].

To assess the hepatoprotective properties of extracts, *in vivo* study were performed to measure the serum markers and chemokine presence in it. According to Koch et al. [33], ethanol catabolism will result in surplus of NADH and acetyl-CoA thus causing lipogenesis of cholesterol and TG and also the leakage of cellular enzymes into plasma associated with serum ALT and AST. These will eventually contribute to liver injury. Thus, by restoring the level of serum ALT, AST, cholesterol, and TG back to normal, high dose of fermented mung beans has certified its hepatoprotective effects at least in part. Moreover, in the present study, hepatoprotective effects of mung bean was compared against germinated and fermented mung beans extracts. Higher degree of reduction in serum ALT, AST, cholesterol, and TG content were observed in germinated and fermented mung bean groups as compared to mung bean groups. Nonetheless, mung bean extracts also contributed to slight decline of those serum biomarkers. Our result was in agreement with previous works done on fermented food products where the induced serum markers were significantly restored back to normal through *in vivo* [36] and *in vitro* [19] studies.

The antioxidant properties of extracts were examined in mice liver tissue via MDA, SOD, FRAP, and NO assay. Increased amount of MDA in ethanol-induced liver signifies the enhance degree of lipid peroxidation, which can lead to liver damage. On contrary, SOD and FRAP levels in ethanol-induced group were decreased. A decrease in both activities in liver tissue of ethanol-induced group was largely due tothe impairment of antioxidant enzymes that safeguard cells against reactive oxygen species [31]. On the other hand, increase in SOD and FRAP levels and decrease in MDA formation in fermented and germinated extracts-treated groups were as expected. High total phenolic content and strong antioxidant activity were claimed in fermented [37, 38] and germinated mung bean [14, 39]. This may be the reason for the increase of SOD and FRAP activities in germinated and fermented mung bean at high dose as compared to mung bean, which consequently reduced the MDA level. In addition, it has been reported that mung bean extract contains volatile antioxidant which was able to inhibit malonaldehyde formation in blood plasma [40].

NO is an inflammatory mediator and highly reactive oxidant produced by iNOS, which is released by kupffer cells upon exposure to hepatotoxins [5, 41]. In all extracts-treated groups, NO level was reverted to normal level. High dose fermented mung bean extracts were the most effective extracts to revert the elevation of NO level after induction with ethanol followed by germinated mung bean extracts. Thus, by suppressing NO production in liver, germinated and fermented mung beans depicted their potential properties as hepatoprotective agent.

A plausible justification for hepatoprotective and antioxidant effects of germinated and fermented mung beans at high dose may be due to the presence of flavonoids and phenolic acids bioactive compounds, which were highly detected particularly in fermented and germinated products [14, 42]. Besides, many studies have reported on the increased content of GABA and amino acids in commercial legumes after undergoing germination [43–46] and fermentation [21, 47] processes. Elevation of amino acids and GABA in our germinated and fermented mung bean extracts may be well added to liver protection properties since GABA amino acids have been known to carry liver protection through the mechanism of maintenance of intracellular polyamines levels of ethanol and CCl_4_-exposed hepatic injury effects [48, 49].

In the present study, histological evaluation was undertaken to support the biochemistry profiles. The pathological changes observed in the ethanol-treated liver through H&E staining were related to the results obtained. Administration of ethanol in mice animal model revealed that elevated level of liver function biomarkers ALT, AST, TG, and cholesterol levels were detected along with the decrease of antioxidant activity and severe necrosis histopathological changes. However, possible hepatoprotective effect of germinated and fermented mung bean extracts was observed when attenuated liver was treated with extracts. Previous study has reported the hepatoprotective properties of their extract to reduce microvesicular steatosis and hepatocytes necrosis in chronic liver injury, which is in agreement with our study [9]. Liver injury hallmark such as inflammation, lymphocytes infiltration, necrosis and ballooning effects were restored back close to normal after administration of high dose of germinated and fermented mung bean extracts-treatment, supported by the decrease in ALT, AST, TG, cholesterol, NO, MDA and increase in FRAP and SOD activities. The correlation between liver biomarkers and histopathological changes suggested that they can be used for early detection of acute liver damage. Reduction of biochemical and histological damage was exerted by fermented and germinated mung bean, conforming their hepatoprotective properties.

No studies have been conducted on histopathological changes of fermented and germinated mung bean extracts on ethanol-attenuated liver. The biochemical and histopathological changes of attenuated liver after being treated with fermented and germinated mung bean were as expected since they contain more bioactive compounds compared to mung bean. Results prove that germinated and fermented mung bean exert better effects on liver injury than normal mung bean. This implies that the increase in amino acids, GABA, phenolic content, and other bioactives compounds during germination and fermentation processes contribute to the hepatoprotective effects of mung bean to ameliorate liver injury. Overall, fermented mung bean possessed the best antioxidant and hepatoprotective effect. This result gives us an idea that amino acid may play a more important role than GABA since we have observed better improvement of amino acid level in fermented than germinated mung bean but vice versa for GABA content.

## 5. Conclusion

To the best of our knowledge, no comparison studies have been made specifically between mung bean and germinated or fermented mung bean aqueous extracts in terms of their liver hepatoprotective and antioxidant enzyme properties. Freeze-dried fermented and germinated mung bean aqueous extracts at 1000 mg/kg body weight showed potential hepatoprotective effects on ethanol-induced liverinjury based on serum biochemical profile and histology evaluation of mice liver. This could be largely due to the amino acids content and antioxidant properties possessed by these extracts with regard to FRAP scavenging activity and oxidant-related factor, SOD. In conclusion, fermentation and germination increased the nutritional and medicinal values of mung bean. Moreover, the results are comparable with silybin, a standard drug typically prescribed to treat liver disease. Therefore, the results strongly imply the potential use of fermented and germinated mung bean aqueous extracts from natural product in future application for oxidative stress and liver disease therapy.

## Figures and Tables

**Table 1:** Effect of mung bean extracts on serum ALT, AST, TG, and cholesterol in alcohol-induced acute liver toxicity in mice.

Treatment	ALT (U/L)	AST (U/L)	TG (mmol/L)	Cholesterol (mmol/L)
Normal untreated	14.09 ± 1.53	98.16 ± 1.99	1.48 ± 0.23	3.14 ± 0.39
50% EtOH (placebo)	48.11 ± 1.78	367.30 ± 1.10	2.37 ± 0.14	3.80 ± 0.20
50% EtOH + silybin (50 mg/kg)	26.72 ± 1.20*	171.70 ± 3.79*	2.77 ± 0.16	4.20 ± 0.36
50% EtOH + mung bean (200 mg/kg)	63.44 ± 2.73*	294.50 ± 6.28*	2.06 ± 0.22*	3.28 ± 0.31*
50% EtOH + mung bean (1000 mg/kg)	28.09 ± 1.32*	234.19 ± 6.87*	2.05 ± 0.44*	3.29 ± 0.41*
50% EtOH + germinated mung bean (200 mg/kg)	57.57 ± 3.60*	308.61 ± 1.33*	2.21 ± 0.06*	3.40 ± 0.01*
50% EtOH + germinated mung bean (1000 mg/kg)	26.34 ± 3.50*	191.93 ± 1.51*	1.84 ± 0.35*	3.13 ± 0.06*
50% EtOH + fermented mung bean (200 mg/kg)	56.26 ± 4.71*	232.48 ± 1.52*	2.26 ± 0.12*	3.18 ± 0.21*
50% EtOH + fermented mung bean (1000 mg/kg)	17.45 ± 1.88*	110.77 ± 6.96*	1.46 ± 0.76*	2.91 ± 0.19*

Values are mean ± SEM of 8 animals each in a group and significantly different from the 50% EtOH (Placebo) (*P < 0.05) by ANOVA and followed by Duncan's multiple range test.

**Table 2:** Effect of mung bean extracts on SOD, MDA, FRAP, and NO levels in liver homogenate of alcohol-induced acute liver toxicity in mice.

Treatment	SOD (U/mg of protein)	MDA (nmol/g of protein)	FRAP (U/mg of protein)	NO (*μ*mol/mg of protein)
Normal untreated	16.58 ± 0.58*	3.02 ± 0.16*	9.40 ± 1.04*	9.97 ± 0.25*
50% EtOH (placebo)	9.17 ± 0.79	7.17 ± 0.17	5.33 ± 0.04	14.72 ± 0.75
50% EtOH + silybin (50 mg/kg)	17.06 ± 0.01*	4.92 ± 0.20*	14.97 ± 0.08*	9.39 ± 2.70*
50% EtOH + mung bean (200 mg/kg)	16.48 ± 2.72*	3.74 ± 0.25*	8.82 ± 0.25*	11.04 ± 0.39*
50% EtOH + mung bean (1000 mg/kg)	17.07 ± 3.77*	3.78 ± 0.33*	5.63 ± 0.01	10.29 ± 0.11*
50% EtOH + germinated mung bean (200 mg/kg)	16.64 ± 0.73*	2.54 ± 0.20*	9.83 ± 0.02*	9.54 ± 0.04*
50% EtOH + germinated mung bean (1000 mg/kg)	17.11 ± 1.26*	2.31 ± 0.26*	5.74 ± 0.02	8.84 ± 0.42*
50% EtOH + fermented mung bean (200 mg/kg)	18.00 ± 0.34*	3.22 ± 0.32*	5.52 ± 0.02	10.78 ± 0.03*
50% EtOH + fermented mung bean (1000 mg/kg)	21.35 ± 0.44*	2.00 ± 0.23*	11.92 ± 0.03*	9.03 ± 0.06*

Values are mean ± SEM of 8 animals each in a group and significantly different from the 50% EtOH (placebo) (*P < 0.05) by ANOVA and followed by Duncan's multiple range test.

## Abbreviations

ALT: Alanine transaminase
AST: Aspartate aminotransferase
GABA: *γ*-amino butyric acid
TG: Triglycerides
NO: Nitric oxide.
